# Study of Medicine: Lessons Learnt from International Medical Students Conference

**DOI:** 10.31729/jnma.4905

**Published:** 2020-05

**Authors:** Prarthana Subedi

**Affiliations:** 1 Nepalese Army Institute of Health Sciences, Sanobharyang, Kathmandu, Nepal

Medical education forms the foundation upon which the Health Care system of Nepal will be built. Ministry of Education, Universities, NMC (Nepal Medical Council) is playing a role in regulating medical education in Nepal. Health Care is developing at an unprecedented rate worldwide; however, medical schools are not evolving to meet the rapidly developing needs of society. Medical Students are the ones who are directly exposed to the medical curriculum and are the quality checks of medical education, so students must take responsibility in curriculum development and take part as active stakeholders to improve medical education in Nepal.

The history of medical education in Nepal is not long. Institute of Medicine started providing a formal MBBS degree from the year 1978.^1^ The NMC recommends that each medical college have their own medical education committee.^1^ It becomes imperative to note that the integrated system of teaching in Nepal was developed adopting the concepts of Mc Master and Maastricht education system.^1^

To further advance the medical teaching, WHO has established Regional Medical Education training centers, however only two centers existed during the time health education started in Nepal in the SEARO (South East Asia Regional Office) region, 1^st^ being in Peradeniya,Srilanka and the 2^nd^ in Chulalongkorn University, Bangkok Thailand.^1^

Attending two conferences for medical students worldwide in Hongkong and Thailand over duration of six months came with a lot of learning and understanding from a student’s perspective, the curriculum of various medical colleges around the world. SCOME (Standing committee on Medical Education), one of the standing committeesof IFMSA (International Federation of Medical Students Association), took forward to express views about medical education and the role of medical students in improving it.

The idea of how I would like to study began forming, and it took shape. Bringing all the lessons back here to Nepal, few points have been mentioned as to what other medical students get to experience in their medical education, and how this could be a promising idea here in Nepal.

## PEER TEACHING PLAYS A KEY ROLE

Didactic lectures have been the mode of delivering content in Medical schools of Nepal. Students tend to learn better when they develop a good relationship with their teachers. Peer-based teaching where non-professionals with a common goal, teach, and learn can be an effective way of learning. A small group of 3-5 students can discuss patient notes, radiographic images, ECG interpretation, and asses each other in an examination format.There was a significant increase in pass rate in peer-based teaching as a per meta-analysis, which showed an increase to 98% from 85% on faculty-based teaching.^2^

## LEARNING AND PRACTICING EVIDENCE-BASED MEDICINE

Teaching evidence to all medical students is imperative. But in Nepal, students tend to read the text given in the textbooks without actually determining where the text is coming from. A way of teaching where research articles are assigned for studying before attending the lecture classes would help a student understand the roots of the text they are studying. 75% of students here find it difficult to perform a literature review, and as per a study done, 50% of students in the medical school of Nepal find difficulty in interpreting research articles.^3^ Research is not taught in the medical school curriculum, and it turns out to be an individual venture of a medical student. Mentoring the students to conduct research can foster their interest in the topic and motivate them to go down the path because many a times what holds the students back is the fear of not knowing what to do.^4^

## MENTORSHIP

The importance of mentorship programs hasn’t been talked about enough. Most of my friends were enrolled in a mentorship program. A program that roots its origin back to the USA has gotten worldwide acceptance. But mentoring programs are not present in medical schools here in Nepal. From a view of a student, grueling through the magnanimous course volume that you need to cover, in a new school, away from home, making new friends can cause a significant breakdown in mental health. A mentor can be the empathetic listener, who guides you through and help you build professionalism, counsel you about your career, foster personal growth, and increase your interest in research and academic careers when you graduate, learning and navigating your way through medical school at your own style and pace.

## RESTRUCTURING THE EXAMINATION SYSTEM

Examination pattern specifically of Tribhuvan University is only of a subjective pattern where short answer questions and essay questions comprise the exam paper but Case-based learning would be more effective for us to brainstorm and think rationally and to gear us forward towards decision making in our professional lives.

## EXCHANGES

The other idea to which my friends from Taiwan, Philippines, Thailand, and Korea were exposed to was exchanges. Massive professional and research exchanges were being provided by the university they were studying in. Global outbreaks, like the Ebola, MERS and COVID-19 as of date has prompted the importance of a global health response.^8^ Exposing us to health care systems from various countries harbors a promise that we as medical students and to be professionals,understand the global health system, get exposed to diseases not commonly encountered in our part of the world like inflammatory bowel disease and also understandtechnological advancements. Equivalence plays a key role here. Some students go for a professional exchange from Nepal, but they are not able to get the equivalence of the rotation from the university. Exchanges makes students change makers and help them make an impact in global health with the relations they form in the course of their studies.

## KEEPING IN TOUCH WITH THE CREATIVE ARTS

Teaching the history of medicine, performing arts, theater, sharing books related to medicine like Siddhartha Mukharjee, Paul Kalinathi, and creating a close community who learn empathy, the art of science, and the science of art is very important to raise empathetic individuals. Research has shown that the empathy levels of medical students tend to plummet by the end of the third year as compared to first-year levels after the death and tragedy that students witness.^7^ So performing arts like theatre, drama, and painting can help us keep in touch with the more humane side of our life.

## ETHICS ETHICS AND MORE ETHICS!

Medical ethics is important to any medical practice. The only medical ethics being taught in Nepal is an hour-long forensic lecture in 3^rd^ year and another 1 hour in the doctor-patient relationship in medical sociology. Many students remain misinformed about, end of life decisions, ethical dilemmas, researchethics, publication ethics, which is required in clinical practice. Though the curriculum mentions these aspects, they are hardly being taught here,whereas, in Hongkong and Japan, they do have a different subject on ethics and ethical practice. In a study done in Nepal about 56% students felt that ethics was inadequately taught here in Nepal.^3^

## CONCLUSIONS

With the experiences gathered from the conferences and the discussions done amongst various medical students, the view of how medicine could be learned optimally by a medical student here in Nepal is presented. As we are going to become health professionals, this is how I would love to learn my medicine, what about you?

## Conflict of Interest:

**None.**
